# Investigation of the Expression, Localization, and Acidosis-Associated Conformational Changes in Connexin 43 in Traumatic Brain Injury with the Development of a Neural Network Model for Assessing Systemic Inflammation

**DOI:** 10.3390/ijms26188855

**Published:** 2025-09-11

**Authors:** Chizaram Nwosu, Evgeniya Kirichenko, Stanislav Bachurin, Mikhail Petrushan, Alexey Ermakov, Rozaliia Nabiullina, Marya Kaplya, Alexander Logvinov, Stanislav Rodkin

**Affiliations:** 1 Research Laboratory “Medical Digital Images Based on the Basic Model”, Department of Bioengineering, Faculty of Bioengineering and Veterinary Medicine, Don State Technical University, Rostov-on-Don 344000, Russiabachurin.rostgmu@gmail.com (S.B.); a.k.logvinov@yandex.ru (A.L.); 2 Wizntech LLC, Rostov-on-Don 344002, Russia

**Keywords:** connexin 43, traumatic brain injury, glial cells, neuron, gap junctions, blood cells, inflammation

## Abstract

Traumatic brain injury (TBI) is one of the most common forms of neurotrauma, accompanied by significant disruptions in neuronal homeostasis and intercellular communication. A key protein involved in these processes is connexin 43 (Cx43), which facilitates the formation of gap junctions in the astrocytic network. In this study, using confocal and immunofluorescence microscopy, ultrastructural analysis, and molecular modeling, we investigated the dynamics of Cx43 expression and structural changes in neuroglia during various post-traumatic periods following TBI. It was shown that in the acute phase, 24 h post-injury, there is a reduction in Cx43 expression, accompanied by apoptotic neuronal degradation, disruption of nuclear NeuN localization, and destruction of cellular ultrastructure. By 7 days post-injury, a significant increase in Cx43 levels was observed, along with the formation of protein aggregates associated with pronounced reactive astrogliosis. Peripheral blood analysis revealed persistent neutrophilia, lymphopenia, and reduced monocyte levels, reflecting a systemic inflammatory response and immunosuppression, which was corroborated by a custom-trained neural network-based computer vision model. Linear regression and correlation analyses further identified a strong positive association between normalized monocyte levels and Cx43 expression, a moderate negative correlation with lymphocytes, and no significant correlation with neutrophils. Using a custom-built computer vision model, we confirmed these hematological trends and detected subtle changes, such as early increases in platelet counts, that were not captured by manual evaluation. The model demonstrated strong performance in classifying common blood cell types and proved to be a valuable tool for monitoring dynamic post-traumatic shifts in blood. Molecular dynamics modeling of Cx43 identified a pH-dependent mechanism of conformational reorganization under post-traumatic acidosis, mediated by the interaction between protonated His142 and Glu103. This mechanism mimics the structural consequences of the pathogenic E103K mutation and may play a critical role in the neurotoxic effects of Cx43 in TBI. These findings highlight the complexity of Cx43 regulation under traumatic conditions and its potential significance as a target for neuroprotective therapy.

## 1. Introduction

Traumatic brain injury (TBI) is one of the leading causes of death and disability worldwide. To date, no clinically effective selective neuroprotective agents exist that can protect neurons and glial cells from traumatic damage. This represents a major challenge for global healthcare systems and necessitates the identification of new therapeutic targets and treatment strategies [1]. One such molecular target is Connexin 43 (Cx43)—the principal gap junction (GJ) protein in the central nervous system (CNS), responsible for the formation of intercellular GJs [2]. This issue becomes particularly relevant in the context of TBI, which induces profound disruptions in neuronal homeostasis and dysregulation of neuroglial interactions [3,4,5].

The duality of Cx43-mediated effects, which may contribute both to neuroprotection and neurotoxicity, makes it a complex subject of investigation and drives the search for the underlying mechanisms behind this functional dualism and associated signaling pathways [6,7,8,9,10].

Cx43 is a transmembrane protein with a molecular weight of 43 kDa, composed of four transmembrane domains, two extracellular loops, and cytoplasmic N- and C-terminal regions. It assembles into hexameric structures—connexons—that can function as hemichannels. When two hemichannels from adjacent cells dock together, they form GJs, enabling direct intercellular exchange of ions and metabolic signals [2,11,12]. The structural and functional organization of Cx43 in the CNS represents a complex spatiotemporal system, underlying its diverse biological effects that contribute to maintaining neural tissue homeostasis both under physiological and pathological conditions.

Disruption of Cx43-mediated intercellular communication is implicated in the pathogenesis of numerous neurological disorders [3]. In the context of TBI, disturbances in this system trigger a cascade of molecular and cellular events that largely determine the fate of damaged neurons and glial cells [8,13]. On one hand, Cx43-based GJs serve as “molecular highways” for the delivery of essential substrates—and even organelles such as mitochondria—to the injury site [12,14]. In addition, astrocytic networks connected via Cx43 form buffering systems that neutralize excess excitotoxins and extracellular potassium ions (K^+^), thereby limiting neuronal hyperexcitability and exerting a neuroprotective function [9,15]. However, excessive activation of Cx43 channels may facilitate the spread of pro-inflammatory cytokines [16], apoptotic signals [17], and calcium waves [18], thereby expanding the area of secondary injury and promoting cell death.

In addition, the immune response may play a crucial role in this dynamic. Leukocytes recruited to the site of injury after TBI not only amplify inflammation through the release of pro-inflammatory mediators but can also directly or indirectly regulate the expression and functional state of Cx43. For example, neutrophil infiltration via the CXC chemokine receptor 2 (CXCR2) axis after TBI [19] is associated with increased Cx43 levels, and pharmacological blockade of CXCR2 reduces both neutrophil activation and Cx43 expression [20]. Monocytes and macrophages migrating to the lesion site enhance pro-inflammatory signaling, including the release of interleukin-1 beta (IL-1β) and tumor necrosis factor alpha (TNF-α), which directly decrease Cx43 expression in astrocytes [21]. Lymphocytes, particularly Th1 cells, participate in maintaining the inflammatory balance and can also indirectly affect astrocytic GJs through IFN-γ-mediated microglial activation and subsequent IL-1β release, which reduces Cx43 levels [22]. The interplay between immune cells and Cx43 forms a critical axis that determines the balance between neuroprotective and neurotoxic outcomes following TBI.

Another pressing issue in the clinical management of TBI is the development of prognostic tools for outcome prediction in severe cases. One promising direction is the analysis of blood-based parameters [23]. Although routine laboratory testing is well established in clinical practice for TBI assessment and monitoring, its diagnostic accuracy remains limited. Many biomarkers and correlates of TBI may be difficult to detect and quantify reliably across different clinical settings. Furthermore, significant changes in the qualitative and quantitative composition of blood following TBI warrant deeper scientific investigation [24].

An additional challenge is the manual counting of certain blood components, such as differential white blood cell counts. This process is time-consuming, labor-intensive, and susceptible to human error, as it heavily depends on the expertise, attentiveness, and experience of the laboratory technician. While automated hematology analyzers can partially address this problem, their high cost—both for initial acquisition and ongoing maintenance—limits their availability, especially in resource-constrained healthcare settings [25].

Moreover, beyond basic indicators such as total leukocyte counts or neutrophil-to-lymphocyte ratios, more nuanced analyses are required. These include investigating specific subpopulations of immune cells, morphological abnormalities, changes in cell size, and other features at different post-traumatic time points. This highlights the need to develop a dedicated computer vision model to automate routine analyses, reduce the workload of medical personnel, and provide more accurate, standardized, and timely clinical assessments for patients with severe TBI.

In our previous studies, we developed machine learning (ML) models for cell counting and identification [26], as well as for the detection of apoptotic nuclear changes induced by severe TBI [27]. In the present study, we combined advanced molecular biology methods with IT technologies to investigate the spatiotemporal dynamics of Cx43 in the brain tissue following severe TBI. We also performed in silico modeling of conformational changes in Cx43 under acidosis—a condition characteristic of the injured brain region—and comprehensively analyzed changes in blood cellular composition in response to traumatic impact, as well as conducted a detailed investigation of changes in peripheral blood cell counts in response to traumatic injury, demonstrating the key role of monocytes in regulating Cx43.

## 2. Results

### 2.1. Spatiotemporal Analysis of Cx43 Expression

**Figure 1:**
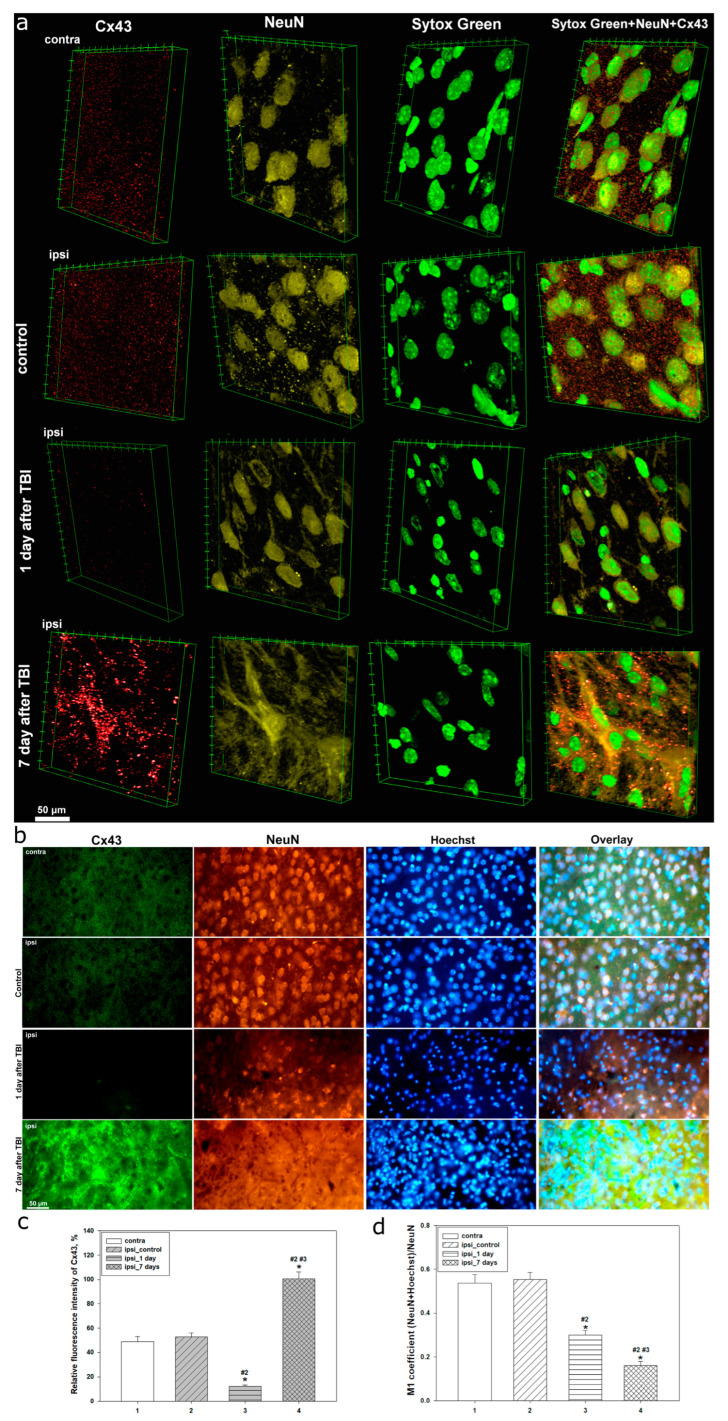
(**a**) Confocal laser scanning microscopy illustrating Cx43 expression in the contralateral and ipsilateral hemispheres of the brain in control and experimental animals at 1 and 7 days post-TBI. Red fluorescent signal indicates immunolabeling of Cx43, dark yellow corresponds to the neuronal nuclear marker NeuN, and green indicates nuclear staining with Sytox Green. Colocalization of the indicated markers is visualized through signal overlay and 3D reconstruction in the final images—Sytox Green, NeuN, Cx43. Scale bar: 50 µm. (**b**) Immunofluorescent microscopy: Cx43 expression (green fluorescence) in the cerebral cortex of control and experimental animals at 24 h and 7 days post-TBI. Contralateral (Contra) and ipsilateral (Ipsi) hemispheres are indicated. NeuN (orange fluorescence) marks neuronal nuclei; Hoechst 33,342 (blue fluorescence) stains the nuclei of all cells, including neurons and glia. The overlay panel shows the merged channels of Cx43, NeuN, and Hoechst. Scale bar: 50 µm. (**c**) Mean fluorescence intensity of Cx43 in the contralateral and ipsilateral cortex of control and experimental animals at 24 h and 7 days post-TBI. (**d**) Colocalization coefficient M1 between NeuN and Hoechst signals in the contralateral and ipsilateral cortex of control and experimental groups at 24 h and 7 days post-TBI. Numbers under bars correspond to group labels: 1—contralateral cortex; 2—control ipsilateral cortex; 3—ipsilateral cortex at 1 day post-TBI; 4—ipsilateral cortex at 7 days post-TBI. * *p* < 0.05 indicates a significant difference between ipsilateral and contralateral cortex; # *p* < 0.05 indicates a significant difference between experimental and control ipsilateral cortex. Significance markers #2, #3 above bars indicate statistically significant differences between corresponding groups. Data are presented as Mean ± SEM. Statistical analysis was performed using ANOVA with Tukey’s post hoc test, *n* = 6, where *n* represents the number of animals in each group.

**Figure 2:**
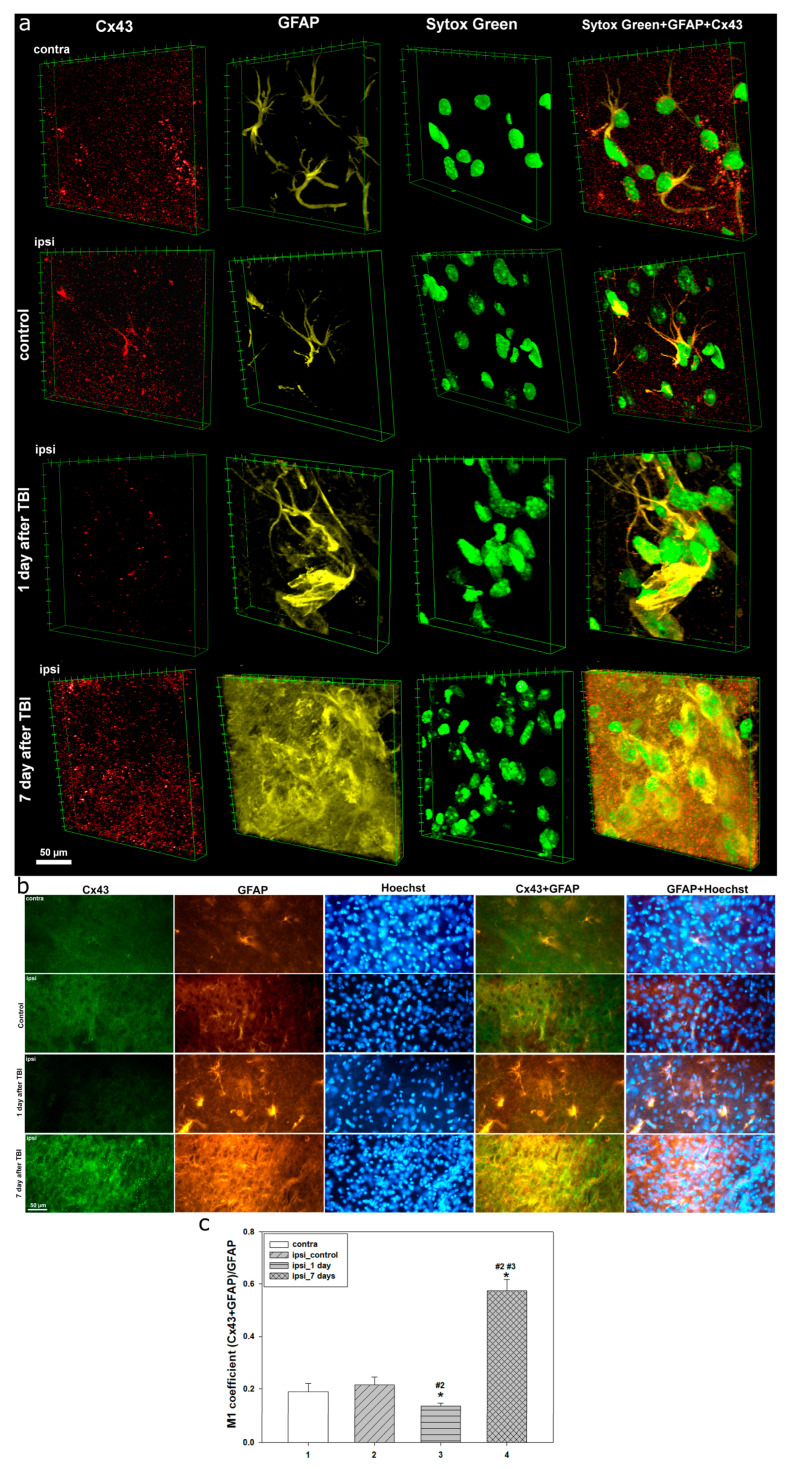
(**a**) Confocal laser scanning microscopy illustrating Cx43 expression in the contralateral and ipsilateral hemispheres of the brain in control and experimental animals at 1 and 7 days post-TBI. Red fluorescent signal indicates immunolabeling of Cx43, dark yellow corresponds to the astrocytic marker GFAP, and green indicates nuclear staining with Sytox Green. Colocalization of the indicated markers is visualized through signal overlay and 3D reconstruction in the final images—Sytox Green, GFAP, Cx43. Scale bar: 50 µm. (**b**) Immunofluorescent microscopy: Cx43 expression (green fluorescence) and its colocalization with GFAP in the cerebral cortex of control and experimental animals at 24 h and 7 days post-TBI. Images show contralateral (Contra) and ipsilateral (Ipsi) hemispheres. GFAP (orange fluorescence) is used as an astrocyte marker; Hoechst 33,342 (blue fluorescence) stains nuclei of all cells, including neurons and glia. The panel Cx43+GFAP displays merged signals of Cx43 and GFAP, and GFAP+Hoechst shows the overlay of GFAP with nuclear stain. Scale bar: 50 µm. (**c**) Colocalization coefficient M1 between Cx43 and GFAP signals in the contralateral and ipsilateral cortex of control and experimental groups at 24 h and 7 days post-TBI. Numbers under bars correspond to group labels: 1—contralateral cortex; 2—control ipsilateral cortex; 3—ipsilateral cortex at 1 day post-TBI; 4—ipsilateral cortex at 7 days post-TBI. * *p* < 0.05 indicates a significant difference between ipsilateral and contralateral cortex; # *p* < 0.05 indicates a significant difference between experimental and control ipsilateral cortex. Significance markers #2, #3 above bars indicate statistically significant differences between corresponding groups. Data are presented as Mean ± SEM. Statistical analysis was performed using ANOVA with Tukey’s post hoc test, *n* = 6, where *n* represents the number of animals in each group.

Confocal microscopy revealed that in the intact cerebral cortex, Cx43 expression exhibits a uniform distribution without signs of aggregation or the formation of consolidated structures. Notably, no differences in the distribution or expression levels of Cx43 were observed in the contralateral hemisphere of both control animals and animals after TBI, as well as in the ipsilateral cortex of the control group (Figure 1a and Figure 2a).

However, as early as 24 h after TBI, significant alterations in the localization and expression levels of Cx43 are observed in the injured area (Figure 1a and Figure 2a).

A near-complete reduction in Cx43 expression is detected, accompanied by pronounced nuclear destruction. Morphological changes detected using Sytox Green include nuclear shrinkage, fragmentation, and chromatin condensation, indicating the activation of cell death mechanisms (Figure 1a and Figure 2a). Concurrently, a decrease in the immunoreactivity of the neuronal marker NeuN is observed in the nucleoplasm, and in some neurons, translocation of NeuN from the nucleus to the perinuclear region is noted (Figure 1).

At this stage, a pronounced glial response also begins to emerge. Under normal conditions, astrocytes display well-defined somata and processes. However, 24 h post-injury, GFAP reactivity is markedly increased. Astrocytic processes become more ramified, and morphologically atypical astrocytes lacking clearly defined processes are more frequently observed (Figure 2a).

Seven days after TBI, the expression of Cx43 shows an opposite trend—a marked increase with heterogeneous distribution, often accompanied by the formation of large aggregates and Cx43 clusters (Figure 1a and Figure 2a). At the same time, NeuN nuclear immunoreactivity continues to decline, reaching critically low levels or even complete absence, indicating ongoing neuronal degradation (Figure 1a). Sytox Green staining, at this time point, shows clear signs of cell death, including karyolysis, pyknosis, and other morphological features characteristic of the late stages of cell death (Figure 1a and Figure 2a). In addition, the astrocytic response is further intensified. In the lesion area, atypical astrocytes lacking processes are frequently observed, and GFAP expression becomes predominantly somatic, localizing to the cell body (Figure 2a).

Immunofluorescent microscopy was used to characterize in detail the distribution and expression of Cx43, as well as to assess alterations in neuronal and astrocytic status in the rat cerebral cortex under normal conditions, and at 24 h and 7 days post-TBI (Figure 1b and Figure 2b). The results reflect the phasic dynamics of the cellular response to traumatic injury.

In intact tissue and in the control group, Cx43 expression in the cerebral cortex was evenly distributed. Cx43 was visualized as homogeneous, fine-granular fluorescence without the formation of aggregates (Figure 1b and Figure 2b), indicating stable functional activity of GJs and preserved physiological astrocytic communication. Neuronal nuclei labeled with NeuN retained clear nuclear localization, characteristic of mature neurons. The nuclei were regularly shaped, with well-defined nucleoli (Figure 1b). Astrocytes identified via GFAP exhibited their characteristic stellate morphology, with long, thin processes and no signs of hypertrophy or reactivity (Figure 2b).

By 24 h post-TBI, significant molecular and morphological disturbances were observed. Cx43 expression decreased more than fourfold in the ipsilateral cortex compared to control (*p* < 0.05) (Figure 1c), with the fluorescent signal becoming weak and diffuse. This reduction was accompanied by neuronal alterations, including a nearly twofold decrease in NeuN levels (*p* < 0.05) (Figure 1d), loss of clear nuclear localization, and translocation of the signal to the perinuclear zone, indicating the activation of pathological intracellular processes. The colocalization coefficient M1 between NeuN and Hoechst decreased by approximately 45% (*p* < 0.05), confirming compromised nuclear integrity and the initiation of degenerative changes (Figure 1d).

Astrocytes in the injured area exhibited signs of reactivity, including increased GFAP signal intensity, hypertrophy of cell bodies, and thickening of processes (Figure 2b). The spatial distribution of astrocytes became less uniform, consistent with glial activation. These changes indicate the onset of reactive gliosis during the acute phase of injury.

By 7 days post-TBI, Cx43 expression significantly increased, exceeding control levels by more than twofold (*p* < 0.05) (Figure 1c). However, instead of a physiologically uniform pattern, large aggregates and clusters of the protein were observed, often linearly aligned along the presumed injury zone (Figure 1a and Figure 2a). This redistribution may reflect active migration and interaction of reactive astrocytes during tissue reconstruction.

The neuronal population exhibited further signs of degeneration. NeuN levels significantly decreased compared to both the control and the 1-day time point (*p* < 0.05), with complete loss of nuclear localization. Colocalization with Hoechst declined more than threefold (*p* < 0.05) (Figure 1d), indicating severe nuclear disorganization and progressive neurodegeneration. Nuclei became deformed, with indistinct contours and features of destruction.

At this stage, astrocytes underwent pronounced reactive transformation, with hyperplasia, enlarged somata, and loss of normal morphology. GFAP-positive astrocytes formed dense clusters in the injured area (Figure 2b), likely contributing to glial scar formation. Their spatial distribution corresponded to areas of Cx43 accumulation, emphasizing the functional interdependence of these processes.

Quantitative analysis of the M1 coefficient of Cx43 and GFAP colocalization revealed pronounced phase-dependent changes in the dynamics of the post-traumatic response. In the intact and ipsilateral control cortex, the values remained low, corresponding to a physiologically normal level of colocalization. However, as early as 1 day after TBI, the coefficient decreased by 29% compared to the contralateral cortex and by 38% compared to the ipsilateral control group (*p* < 0.05) (Figure 2c). This indicates a marked weakening of the interaction between Cx43 and GFAP, reflecting the initial destabilization of astrocytic gap junctions.

At 7 days after TBI, the opposite trend was observed: the M1 coefficient increased almost threefold compared to control (*p* < 0.05). Moreover, this value was 4.3 times higher than that observed at 1 day post-TBI (*p* < 0.05) (Figure 2c).

### 2.2. Ultrastructural Alterations in Neural Tissue Following Severe TBI

**Figure 3:**
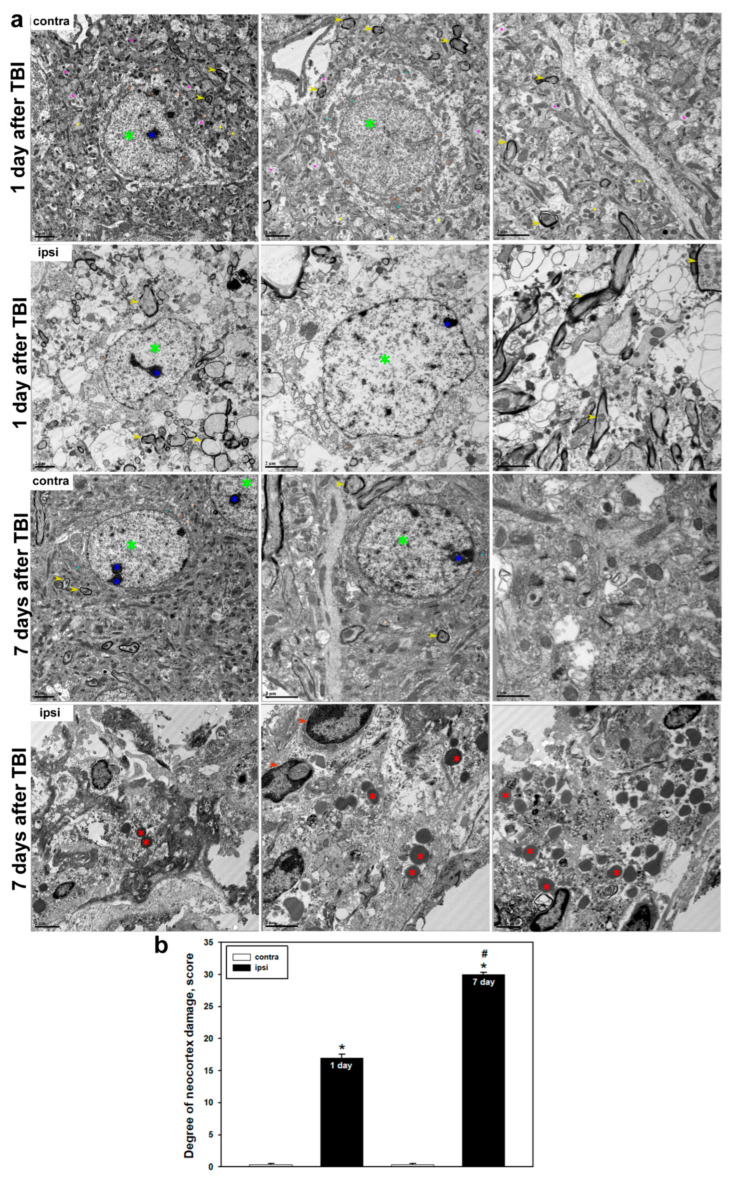
(**a**) Transmission electron microscopy of the cerebral cortex at 1 and 7 days post-TBI. Ultrastructural images are shown for the contralateral (uninjured) and ipsilateral (injured) hemispheres. Color coding: green star—neuronal nucleus; blue star—nucleolus; orange star—mitochondria; light blue star—rough endoplasmic reticulum; beige star—dendritic spines; yellow star—unmyelinated axons; pink star—dendrite; red star—lysosomes. Yellow arrow indicates a myelinated fiber; red arrow indicates a microglial cell. (**b**) Quantitative representation of ultrastructural damage in the cortex, calculated using a semiquantitative scoring system based on eight morphological criteria. Data are presented as Mean ± SEM. Statistical analysis was performed using ANOVA followed by Tukey’s post hoc test; * *p* < 0.05 indicates a significant difference between ipsilateral and contralateral cortex; # *p* < 0.05 indicates a significant difference between ipsilateral at 1 and 7 days after TBI.

Ultrastructural analysis revealed profound disruptions in the architecture of neural tissue induced by TBI, which progressively worsened during the post-traumatic period. In the uninjured (contralateral) hemisphere, no pathological alterations were observed. Neurons retained their normal morphology with intact plasma membranes, and chromatin within the nuclei was evenly distributed, with visible nucleoli. The cytoplasm contained numerous free ribosomes, and mitochondria were present in both the neuronal soma and processes. Well-preserved structures of the rough endoplasmic reticulum (rER) and the Golgi apparatus were clearly distinguishable. Dendrites and axons maintained well-defined structures, showing no signs of edema or vacuolization. The myelin sheath of nerve fibers appeared dense and uniform, without evidence of degeneration (Figure 3a).

However, by 24 h post-TBI, pronounced pathological changes were already evident. Neuronal plasma membranes appeared critically thinned or disrupted, and the nuclear envelope showed signs of degradation. The cytoplasm exhibited severe disorganization, with fragmentation of intracellular compartments. The number of mitochondria in the soma and processes was markedly reduced, and the remaining organelles showed clear signs of damage, including cristae loss, swelling, and deformation. The myelin sheath of axons displayed discontinuities, with areas of delamination, tearing, and cavitation. Severe impairment of axonal transport was also observed, manifested by accumulation of vesicles and disrupted organelles within axonal segments. In some cases, neurons were found to be completely destroyed (Figure 3a). These changes were accompanied by a statistically significant increase in morphological damage compared to the contralateral side, which remained intact (*p* < 0.05) (Figure 3b).

By 7 days post-TBI, destructive changes had critically progressed and became diffuse. Neural tissue had almost entirely lost its normal ultrastructural organization. Cellular architectural elements were either absent or present only as remnants. The tissue exhibited hallmarks of irreversible injury. In addition, numerous amoeboid microglial cells were observed, consistent with phagocytic activation. Their cytoplasm contained abundant lysosomes, phagolysosomes, and inclusions with degraded cellular debris (Figure 3a). Quantitative analysis confirmed a further increase in injury severity: the mean damage score in the injured hemisphere approached maximum values, whereas the contralateral side remained minimally affected (*p* < 0.05) (Figure 3b). A statistically significant difference was also observed between day 1 and day 7 post-TBI within the injured region (*p* < 0.05) (Figure 3b).

### 2.3. Changes in Peripheral Blood Cell Composition Following TBI

**Figure 4:**
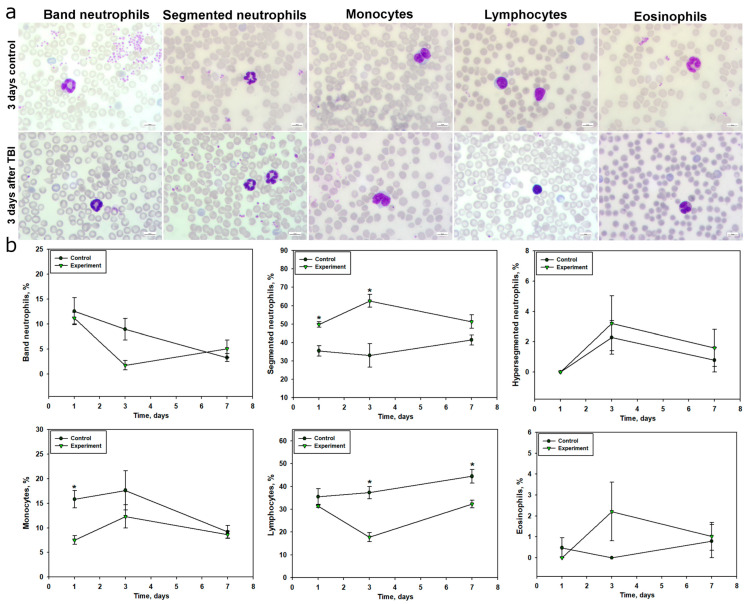
Changes in peripheral blood leukocyte composition following TBI. (**a**) Blood smear microscopy stained with Romanowsky–Giemsa in control (sham-operated) and experimental (TBI) groups. Representative images show banded and segmented neutrophils, monocytes, lymphocytes, and eosinophils. Scale bar: 10 µm. (**b**) Dynamics of leukocyte population changes in peripheral blood on days 1, 3, and 7 post-TBI in control and experimental groups. Data are presented as Mean ± SEM, *n* = 6, where *n* represents the number of animals in each group. Statistical analysis was performed using repeated-measures ANOVA with Holm’s post hoc test. * *p* < 0.05 indicates statistically significant differences between experimental and control groups.

Analysis of peripheral blood in mice after TBI revealed pronounced alterations in leukocyte profiles compared to the control group (Figure 4). Already at 24 h post-TBI, there was a significant increase in the number of segmented neutrophils by 40.1% compared to controls (49.79% vs. 35.54%; *p* < 0.05) (Figure 4), indicating a rapid onset of acute inflammatory response. On day 3, the proportion of these cells increased even further—by 89.5% relative to controls (62.65% vs. 33.05%; *p* < 0.05), reflecting an intensification of neutrophilic reactivity. On day 7, segmented neutrophils also remained elevated by 24% (51.35% vs. 41.41%) compared to the control group (Figure 4b, Table 1).

In contrast, the dynamics of banded neutrophils followed a different pattern. On day 3, their proportion in the TBI group decreased almost fivefold compared to controls (1.80% vs. 8.97%). By day 7, a moderate increase of 54% was observed (5.12% vs. 3.32%) (Figure 4b, Table 1).

Monocyte levels in the TBI group dropped by 2.1-fold at 24 h compared to controls (7.54% vs. 15.83%; *p* < 0.05), possibly reflecting cell redistribution or migration to the injury site. On day 3, monocyte levels remained 30% lower (12.34% vs. 17.63%), and by day 7, the difference had decreased to 6% (8.62% vs. 9.2%) (Figure 4b, Table 1).

The dynamics of lymphocyte counts showed a significant reduction in the TBI group compared to controls beginning at day 3 post-injury. For example, on day 3, lymphocyte levels were reduced by 52.3% (17.79% vs. 37.3%; *p* < 0.05), and by day 7, the decrease persisted at 27.4% (32.29% vs. 44.48%; *p* < 0.05). These changes may reflect a systemic suppression of the adaptive immune response in the context of injury-induced inflammation (Figure 4b, Table 1).

Eosinophils remained scarce at all time points. At 24 h post-TBI, they were absent in the experimental group (0.0% vs. 0.48% in controls; *p* > 0.05). However, by day 3, their proportion increased to 2.21% vs. 0.0%. On day 7, a moderate elevation of 29% was observed (1.02% vs. 0.79%; *p* > 0.05) (Figure 4b, Table 1).

**Figure 5:**
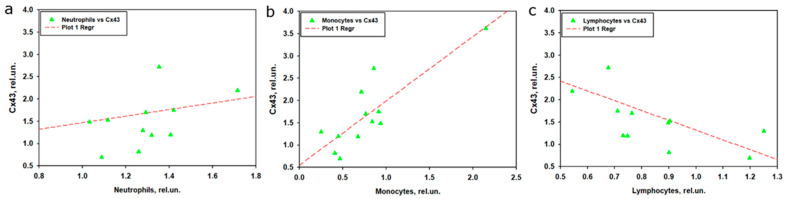
Linear regression analysis of normalized Cx43 expression levels as a function of normalized peripheral blood leukocyte subset levels on days 1 and 7 post-(TBI). (**a**) Neutrophils: the model explains 46.8% of Cx43 variability (R^2^ = 0.468, adjusted R^2^ = 0.350, F(2,9) = 3.965, *p* = 0.058), with an insignificant contribution from neutrophils (β = 0.519, *p* = 0.647) but a significant effect of time (β = 1.052, *p* = 0.023). (**b**) Monocytes: the model accounts for 75.4% of Cx43 variability (R^2^ = 0.754, adjusted R^2^ = 0.700, F(2,9) = 13.83, *p* = 0.002), with a strong positive contribution from monocytes (β = 1.182, *p* = 0.009), while time is insignificant (β = 0.397, *p* = 0.259). (**c**) Lymphocytes: the model explains 52.7% of Cx43 variability (R^2^ = 0.527, adjusted R^2^ = 0.422, F(2,9) = 5.018, *p* = 0.034), but neither lymphocytes (β = −1.207, *p* = 0.271) nor time (β = 0.836, *p* = 0.073) are significant predictors. Data are presented for *n* = 12 (6 values per day).

To investigate the relationship between Cx43 expression and the levels of neutrophil, monocyte, and lymphocyte subpopulations in peripheral blood after TBI, linear regression and correlation analyses were performed using normalized data at 1 and 7 days post-injury. Linear regression included time and the respective leukocyte subpopulation as predictors, with Cx43 as the dependent variable (Figure 5).

The model for neutrophils explained 46.8% of the variability but did not reach significance (R^2^ = 0.468, adjusted R^2^ = 0.350, F(2,9) = 3.965, *p* = 0.058). Time was a significant predictor (β = 1.052, t = 2.734, *p* = 0.023), while neutrophil levels were not (β = 0.519, standardized β = 0.116, t = 0.474, *p* = 0.647), suggesting that Cx43 dynamics depended on time rather than neutrophil levels (Table 2, Figure 5a).

The model for monocytes demonstrated a strong and significant association, explaining 75.4% of the variability in Cx43 levels (R^2^ = 0.754, adjusted R^2^ = 0.700, F(2,9) = 13.83, *p* = 0.002). Monocyte levels were a significant predictor (β = 1.182, standardized β = 0.692, t = 3.313, *p* = 0.009), indicating a positive association whereby higher monocyte levels were linked to increased Cx43 expression, whereas time had no significant effect (β = 0.397, t = 1.204, *p* = 0.259) (Table 2, Figure 5b).

The model for lymphocytes explained 52.7% of the variability and reached significance (R^2^ = 0.527, adjusted R^2^ = 0.422, F(2,9) = 5.018, *p* = 0.034), but neither time (β = 0.836, t = 2.030, *p* = 0.073) nor lymphocyte levels (β = −1.207, standardized β = −0.305, t = −1.171, *p* = 0.271) were significant predictors, indicating only a weak relationship with Cx43 (Table 2, Figure 5c).

Additionally, Pearson, Spearman, and Kendall correlation analyses were performed to assess pairwise associations between normalized Cx43 levels and leukocyte subpopulations. Monocyte levels showed a strong positive correlation with Cx43 (Pearson: r = 0.846, *p* < 0.001; Spearman: ρ = 0.727, *p* = 0.010; Kendall: τ = 0.485, *p* = 0.031), supporting the regression results. Lymphocytes demonstrated a moderate negative correlation with Cx43, significant in nonparametric tests (Spearman: ρ = −0.692, *p* = 0.016; Kendall: τ = −0.515, *p* = 0.021) but not in Pearson’s test (r = −0.557, *p* = 0.060), which may reflect nonlinearity or the limited sample size. Neutrophils showed no significant correlation with Cx43 (Pearson: r = 0.164, *p* = 0.611; Spearman: ρ = 0.336, *p* = 0.287; Kendall: τ = 0.273, *p* = 0.250), consistent with regression analysis (Table 3).

Correlation between leukocyte subpopulations revealed a moderate negative association between lymphocytes and neutrophils (Pearson: r = −0.608, *p* = 0.036; Spearman: ρ = −0.727, *p* = 0.010; Kendall: τ = −0.576, *p* = 0.009), whereas associations between monocytes and other subpopulations were nonsignificant. These results indicate that among leukocyte subpopulations, monocytes exhibit the strongest positive relationship with Cx43 expression after TBI, likely reflecting their role in inflammatory or intercellular signaling processes, while neutrophils and lymphocytes demonstrate weaker associations (Table 3).

### 2.4. Changes in Peripheral Blood Cell Composition After TBI According to Computer Vision Analysis

**Figure 6:**
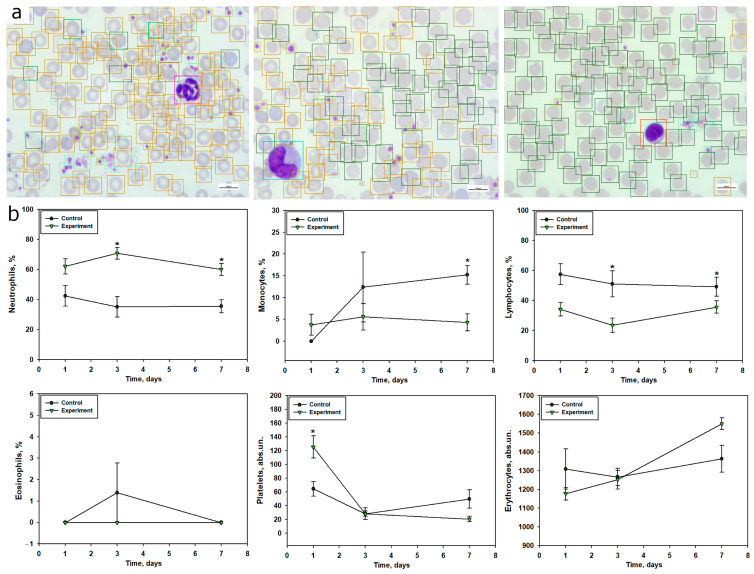
Changes in peripheral blood cell composition following TBI assessed via computer vision. (**a**) Representative micrographs showing automated detection of blood cells. Color coding: neutrophils—pink boxes, monocytes—blue, lymphocytes—red, normal erythrocytes—orange, schistocytes—bright green, platelets—yellow (small boxes), hyperchromic erythrocytes—dark green, artifacts—light green (small boxes). Scale bar = 10 µm. (**b**) Dynamics of leukocyte composition in peripheral blood at days 1, 3, and 7 post-TBI in control and experimental groups. Data are presented as Mean ± SEM, *n* = 6, where n represents the number of animals in each group. Statistical analysis was performed using repeated-measures ANOVA with Holm’s post hoc test. * *p* < 0.05 indicates statistically significant differences between experimental and control groups.

In addition to manual counting, a computer vision model was developed and implemented for automated detection and quantitative assessment of peripheral blood cells, including leukocytes, erythrocytes, and platelets (Figure 6a). The model enabled the recognition of various cell morphotypes, such as normal erythrocytes, schistocytes, hyperchromic erythrocytes, microplatelets, and potential artifacts. Furthermore, the system was capable of distinguishing neutrophil subpopulations—segmented, banded, and hypersegmented forms—although for the purpose of quantitative analysis, all were grouped into a general category of “neutrophils.” Similarly, normal, schistocytic, and hyperchromic erythrocytes were combined into a single erythrocyte population, due to insufficient model accuracy at the current training stage to support reliable subclassification.

The DINO-SwinL was developed to quantify peripheral blood cells in TBI mice, achieving high concordance with manual counts. The model was evaluated on a test set comprising 18 images (10% of the dataset), with annotations validated by one expert, resolving discrepancies through consensus review. Performance metrics included F1-scores of 0.75 for neutrophils (precision: 0.78, recall: 0.72), 0.69 for lymphocytes (precision: 0.72, recall: 0.66), 0.61 for monocytes (precision: 0.65, recall: 0.57), and 0.43 for eosinophils (precision: 0.48, recall: 0.39). The mean average precision (mAP) across all cell types was 0.55, indicating robust detection of common cell types but reduced accuracy for rare populations like eosinophils due to class imbalance.

Due to class imbalance, with rare neutrophil subpopulations (e.g., hypersegmented forms, <1% of annotations) and eosinophils (<0.5% of annotations) underrepresented, these were grouped into broader categories (neutrophils and erythrocytes) for quantitative analysis. This ensured reliable detection, though subclassification accuracy for rare morphotypes was limited (F1-score < 0.60). The model’s results closely mirrored manual counts, confirming its utility for high-throughput analysis of peripheral blood cell dynamics in TBI.

On day 1 post-TBI, there was a 47% increase in neutrophils and a 41% decrease in lymphocytes relative to control values. Additionally, monocytes, which were absent in the control group, became detectable (Figure 6b, Table 4). A statistically significant difference was also observed in platelet count, which nearly doubled (*p* < 0.05). In contrast, the absolute erythrocyte count decreased by 10% compared to controls (Figure 6b).

More pronounced alterations in the leukocyte profile were observed on day 3 post-TBI. Neutrophil counts more than doubled (*p* < 0.05), while lymphocyte levels dropped by 55% (*p* < 0.05). Monocyte numbers were also reduced by 55% compared to controls. Eosinophils were virtually undetectable in both groups (Figure 6b, Table 4). Platelet and erythrocyte counts remained unchanged relative to controls (Figure 6b).

On day 7 post-injury, neutrophil levels remained elevated by 69% (*p* < 0.05), lymphocytes were reduced by 28% (*p* < 0.05), and monocyte numbers dropped by 71% compared to the control group (*p* < 0.05). Eosinophils were not detected in either group (Figure 6b, Table 4). At this stage, platelet counts decreased by 58%, while erythrocyte levels increased by 14% (Figure 6b).

### 2.5. Molecular Dynamics Simulation of Cx43

**Figure 7:**
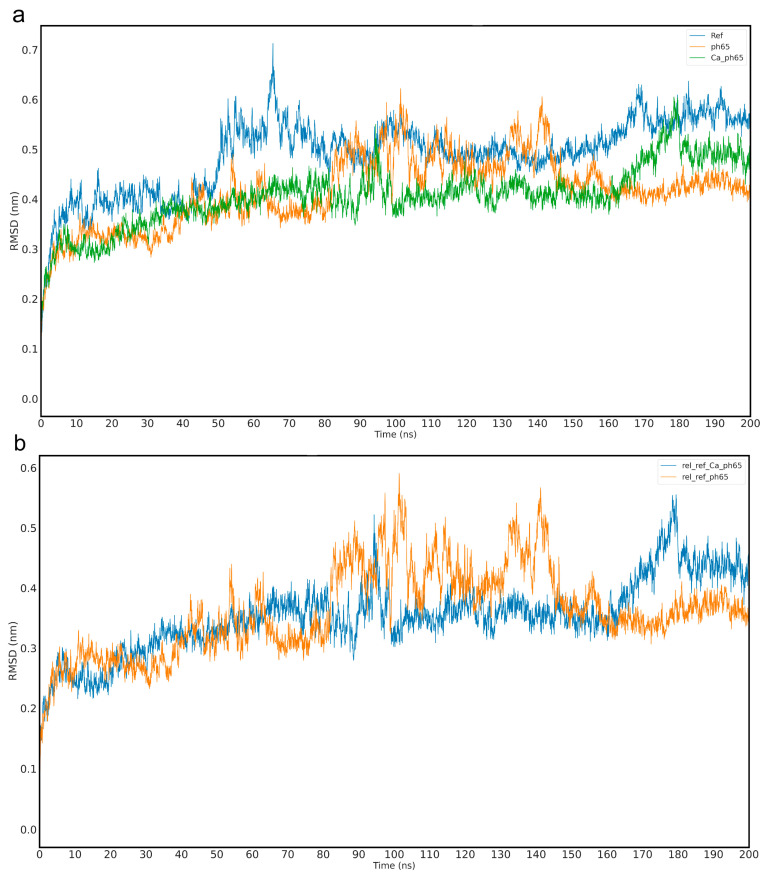
(**a**) RMSD plot of Cx43 geometry under different conditions: Ref—physiological conditions (blue); ph65—ischemic model with pH lowered to 6.5 (orange); Ca_ph65—ischemic model with added Ca^2+^ ions (green). (**b**) Relative RMSD of Cx43 in different media, showing structural deviations at pH 6.5 and pH 6.5 with Ca^2+^ compared to the equilibrium geometry of Cx43 under physiological conditions: ref_ref_pH65—comparison with ischemic model at pH 6.5 (orange); ref_ref_Ca_pH65—comparison with ischemic model at pH 6.5 with added Ca^2+^ ions (blue).

Three histidine (His) residues were identified in the Cx43 protein—His142, His194, and His331—which are susceptible to protonation under acidic conditions (pH 6.5). Two of these residues are located in cytoplasmic regions, while His194 is positioned within an extracellular domain. A 200-nanosecond molecular dynamics (MD) simulation was conducted to evaluate the conformational behavior of Cx43 under various conditions, including a physiological reference state, an ischemic model with reduced pH, and a low-pH model in the presence of Ca^2+^ ions. Throughout the simulation, the geometric stability of the Cx43 molecule was maintained, with RMSD deviations not exceeding 1.0 nm, indicating the absence of major conformational fluctuations that could impact the protein’s biochemical function in the neuronal microenvironment (Figure 7a).

To further evaluate subtle structural rearrangements, an additional plot was generated, illustrating the relative RMSD dynamics of Cx43 at pH 6.5 and at pH 6.5 with Ca^2+^ ions, calculated with respect to the equilibrium geometry under physiological conditions. These data demonstrate that although acidification leads to measurable distortions in the three-dimensional architecture of the Cx43 subunit, it does not induce significant conformational rearrangements, as the RMSD coefficient remains below 1.0 (Figure 7b).

Structural analysis revealed two stable phenomena characterizing Cx43 behavior under pathophysiological conditions. First, the presence of Ca^2+^ ions had no direct impact on the conformation of Cx43. During the simulation, Ca^2+^ ions preferentially interacted electrostatically with phosphate groups of the lipid bilayer, remaining mostly on the membrane surface and forming no stable contacts with Cx43.

**Figure 8:**
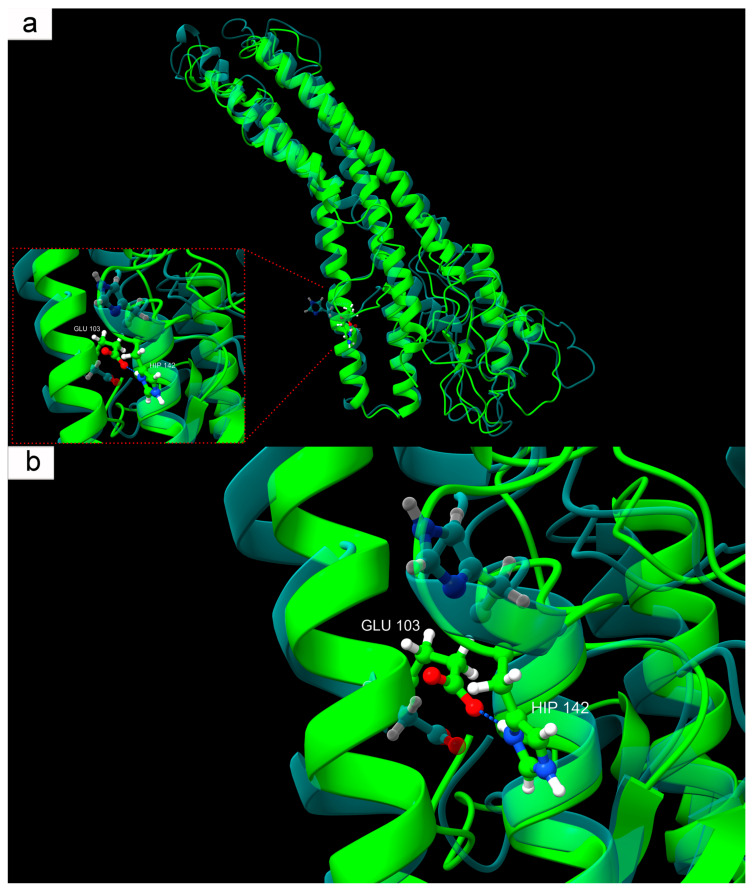
(**a**) Overall structure of Cx43 highlighting the region where a hydrogen bond forms between HIP142 and Glu103. (**b**) Close-up view of the hydrogen bond between HIP142 and Glu103. Color scheme: solid green—Cx43 at pH 6.5; transparent cyan—Cx43 at physiological pH. Structural overlay demonstrates conformational shifts induced by environmental acidification.

The most notable structural behavior was associated with His142. Under physiological pH, this residue did not participate in specific interactions. However, when protonated (HIP142)—as would occur under ischemic acidosis—His142 formed a hydrogen bond with Glu103. This interaction was absent under normal pH: in the reference model, the carboxyl group of Glu103 is oriented toward the aromatic nitrogen of the unprotonated histidine, which contains a lone electron pair, resulting in electrostatic repulsion between similarly charged fragments. This modeling results suggest that local pH shifts can induce subtle but functionally significant conformational rearrangements (Figure 8, Supplementary Materials). Such microtransformations are known to play a critical role in regulating protein–protein interactions, especially in the context of inter-subunit assembly and modulation by regulatory domains.

## 3. Discussion

TBI represents the most common form of neurotrauma and is associated with significant disruptions of neuronal homeostasis. These disturbances manifest as impaired intercellular communication, ionic imbalance, oxidative stress, acute neuroinflammation, ischemia, and other pathological processes [1,28]. Cx43 plays a central role in these events, being a key component of intercellular communication in the CNS by forming GJs [2]. Under neuronal stress caused by TBI, Cx43 largely determines cell fate, promoting either survival or cell death [8,12,13,29]. However, the molecular mechanisms regulating Cx43 expression following TBI remain poorly understood and require further investigation.

Our studies using confocal and immunofluorescent microscopy showed that Cx43 expression is a dynamic system with marked spatial heterogeneity, closely linked to morphofunctional disturbances in neuronal and astrocytic populations of the cerebral cortex during different post-traumatic phases. In intact brain tissue, Cx43 was evenly distributed, reflecting normal physiological activity of GJs and stable intercellular communication within the astrocytic network. Preservation of normal Cx43 levels in the uninjured hemisphere at different time points after TBI is consistent with previous findings [30].

However, as early as 24 h post-TBI, we observed a sharp decrease in Cx43 expression in the injured area, accompanied by severe nuclear damage and a reduction in nuclear localization of the neuronal marker NeuN. This time point corresponds to the acute phase of injury, characterized by apoptosis and necrosis. A decline in NeuN colocalization with the nuclear dye Hoechst indicates significant nuclear permeability disturbances, reflecting deep neuronal degradation. NeuN is a well-established nuclear marker of mature neurons, but its localization has been reported to vary or even be absent under several pathological and physiological conditions [31]. Degradation of differentiated neurons is also often associated with decreased NeuN expression [32]. For example, NeuN levels decline after severe brain injury [33,34], and nuclear-to-cytoplasmic redistribution of NeuN has been reported in models of controlled cortical impact [35]. Fragmented Hoechst- or Sytox Green-positive nuclei were also noted, indicative of TBI-induced apoptotic processes in damaged neural tissue. This observation aligns with our previous study showing marked nuclear fragmentation 24 h after TBI [27].

The decrease in Cx43 may be attributed both to the loss or damage of astrocytes, which are the main source of this protein, and to its degradation under injury conditions. Severe TBI has been shown to reduce Cx43 expression [36]. In models of intracerebral hemorrhage, reduced Cx43 expression was noted 24 h post-injury [37]. Furthermore, Cx43 mRNA levels can decline as early as 2 h after TBI [10], and its protein expression drops immediately after penetrating brain injury [38]. Similar early reductions in Cx43 levels have been reported in several studies [39]. However, other reports showed no change in total Cx43 or p-Cx43 in the injured cortex within 2–8 h post-TBI, whereas p-Cx43 expression increased in the hippocampus, possibly contributing to the spread of secondary damage [13]. Elevated p-Cx43 levels may persist up to 24 h post-injury [30], and it is often considered a neurotoxic astrocytic factor [30,40], associated with abnormal GJ function, whereas total Cx43 is generally linked to neuroregenerative processes [41]. These findings underscore the complex regulatory mechanisms of Cx43 expression in the post-traumatic brain, which depend on numerous factors, including injury severity, type, and location.

The early decline of Cx43 may also be related to the rapid increase in pro-inflammatory cytokines after TBI. It is known that cytokine levels rise sharply within the first few hours following injury [42]. For instance, IL-1β, released by activated microglia, has been shown to downregulate Cx43 expression in astrocytes, as do high concentrations of IFN-γ and IL-17 [22,43]. Notably, the peak pro-inflammatory response occurs within the first 48 h after TBI [44]. Loss of Cx43 function leads to impaired GJ communication, which may exacerbate inflammation, reduce cellular stress resistance, and impair tissue regeneration [2]. Additionally, disrupted intercellular connectivity interferes with ionic and metabolic homeostasis, worsening secondary brain injury.

TBI also initiates neuroinflammatory processes mediated by astrocyte activation. By 24 h post-injury, increased GFAP expression and morphological changes in astrocytes indicate the onset of reactive astrogliosis and functional remodeling aimed at limiting lesion spread. In our study, no prominent astrogliosis was detected, possibly due to astrocyte loss caused by TBI. This is consistent with previous research showing that astrocyte death occurs in the early post-traumatic period, particularly at 24 h [45]. Nevertheless, a moderate early astrocytic response was recorded. Traumatic injury triggers phenotypic changes in astrocytes toward a reactive state, characterized by cellular hypertrophy and thickened processes [46]. Astrocyte proliferation begins within 24 h and peaks by day 3 post-TBI [47]. Such a response may have neuroprotective or cytotoxic effects, depending on injury severity and stage [48].

Seven days after TBI, Cx43 expression showed the most pronounced changes, significantly exceeding control values. Notably, Cx43 distribution became highly uneven, with aggregate formation, likely reflecting disrupted Cx43 functionality amid progressive reactive astrogliosis. These Cx43 plaques may result from excessive protein synthesis and impaired degradation pathways. Similar dynamics have been reported in hypoxia/ischemia models, where peak Cx43 levels occurred on day 7 post-injury [49]. In intracerebral hemorrhage, Cx43 expression decreased at 24 h, increased by day 3, and peaked on day 7, then gradually returned to baseline by two weeks post-injury [37].

At this stage, a further decrease in nuclear NeuN levels and its colocalization with Hoechst was observed, indicating the progression of structural and functional impairments in neurons. The morphology of neuronal nuclei exhibited signs of irreversible degradation, including karyopyknosis and karyolysis, confirming the advancement of cell death processes. These findings are consistent with previous studies showing that the number of pathophysiologically altered cells in injured brain tissue increases over time, starting from the first hours and peaking several days after TBI [50,51]. It has been noted that there is regional variability in the dynamics of degenerating neuronal cells following TBI, but the most pronounced degenerative changes typically occur a few days post-injury [52]. In our previous research, we also demonstrated that TUNEL-positive neurons peak at day 7 after TBI [53].

During this period, reactive astrogliosis becomes more prominent, extending throughout the entire lesion area. Astrocytes undergo significant morphological reorganization, characterized by hyperplasia, loss of the typical stellate shape, and the formation of clusters. These changes indicate the involvement of astrocytes in glial scar formation. This process can represent both a protective and detrimental event in the cascade of molecular and cellular reactions triggered by TBI. While the glial scar serves to contain the spread of secondary injury after severe TBI, it is also associated with negative remodeling of damaged tissue, hindering functional recovery [54]. Interestingly, we observed that Cx43 clusters localize at the borders of GFAP-positive zones, indicating a synchronized astrocytic response to injury aligned with the dynamics of Cx43 expression. In a photothrombotic stroke model, Cx43 was identified as a key player in glial scar formation, being highly expressed in reactive astrocytes within the lesion zone [55]. An increase in Cx43 expression between days 6 and 15 has also been reported in a penetrating TBI model. Notably, the degree of astrogliosis and microglial activation was higher in Cx43 knockout mice, suggesting that Cx43 may exert neuroprotective effects under traumatic neuronal stress [39]. The elevated expression of Cx43 in the late post-traumatic period likely reflects either a compensatory or pathological response aimed at restoring neuronal homeostasis, accompanied by enhanced glial remodeling and fibrotic changes.

Ultrastructural analysis confirmed the presence of pronounced and progressive structural damage in the cerebral cortex following TBI. In the uninjured hemisphere, typical tissue organization was preserved: neurons displayed intact membranes, nuclei with clearly defined nucleoli and evenly distributed chromatin, as well as well-developed ultrastructural organelles, including mitochondria, endoplasmic reticulum, and the Golgi apparatus. Myelin sheaths remained uniform and compact. However, 24 h post-TBI, significant cellular structural disruptions were observed, including membrane and organelle destruction. Mitochondrial degradation was particularly notable, characterized by loss of cristae, swelling, and deformation, indicating energetic dysfunction and activation of cell death pathways. It is well established that mitochondrial dysfunction is a key component of TBI pathogenesis and correlates with injury severity [56]. We also observed myelin sheath destruction, synaptic loss, and impaired axonal transport, indicative of profound neurodegenerative processes. Previous studies have reported that myelin degradation can begin within hours after TBI and progress over time, peaking several days post-injury [57].

By day 7 post-TBI, destructive changes reached a critical level. The cellular architecture was almost entirely lost, with fragmented membranes, disappearance of organelles, and extensive myelin disruption and demyelination. Some cells were completely absent, indicating completion of the cell death process. Quantitative analysis confirmed a significant increase in morphological damage compared to earlier post-traumatic time points. At the same time, we observed accumulation of amoeboid microglial cells containing numerous lysosomes and phagolysosomes, reflecting their active role in debris clearance and tissue remodeling. In the controlled cortical impact model, large-scale neurodegenerative changes were documented in the injured area by day 7, including degradation of axons, dendrites, and intracellular compartments [35]. Other studies have shown that total cytoskeletal destruction of axons, along with diffuse splitting and degradation of myelin, occurs on day 7 following TBI [57].

TBI was accompanied by a pronounced systemic inflammatory response, reflected in the dynamics of peripheral blood leukocyte composition. Within 24 h post-injury, a significant increase in segmented neutrophils was observed, indicating an acute neutrophilic response. This parameter peaked on day 3 and remained elevated by day 7, corresponding to a prolonged phase of systemic inflammation. These findings align with previous studies showing that TBI leads to a significant increase in neutrophil counts within the first 24 h, peaking on day 3, with subsequent normalization by day 7 [58]. Activation of the neutrophilic component of the immune system in the early post-traumatic period after TBI is also supported by several studies [58,59]. Additionally, the increase in neutrophil levels in TBI may result from stress-induced hyperexpression of catecholamines and glucocorticosteroids [60]. The dynamics of band neutrophils indicated a redistribution of mature and immature forms in response to trauma, with a sharp decrease in their levels by day 3, likely reflecting their active migration to the site of inflammation and a high degree of differentiation.

Blood monocyte levels significantly decreased, particularly at 24 h post-TBI, which may be associated with their active migration into damaged brain tissue, where they transform into macrophage-like cells involved in phagocytosis and inflammation regulation [61]. It is known that activated microglia generate pro-inflammatory cytokines, recruiting monocytes and neutrophils from the bloodstream to the damaged area in TBI, a process that can occur in the early post-injury period [62]. Studies have also shown that TBI induces a sharp reduction in monocytes within 24 h post-injury, persisting for up to a month [63].

The most significant and sustained change was a reduction in lymphocyte levels, particularly pronounced on days 3 and 7 post-injury. This indicates suppression of adaptive immunity, characteristic of immunosuppression in severe trauma, increasing the risk of secondary infectious complications due to immune system imbalance. These findings are consistent with previous studies demonstrating that TBI is a potent trigger of long-term lymphopenia [58]. The neutrophil-to-lymphocyte ratio (NLCR) is considered an informative marker for assessing TBI severity and predicting survival outcomes. For instance, a high NLCR correlates with unfavorable outcomes in TBI [64].

Changes in eosinophil levels were transient and likely did not play a key role in TBI pathogenesis, although their increase on day 3 may indicate activation of additional inflammatory pathways.

Using the computer vision model, we confirmed results obtained through manual methods and, in some cases, provided a more detailed analysis of quantitative blood changes in TBI. For example, within 24 h post-TBI, a 47% increase in neutrophil counts relative to the control group was recorded, consistent with manual counts, alongside a 41% reduction in lymphocytes, likely indicating the onset of early TBI-induced immunosuppression. By day 3 post-injury, automated analysis confirmed pronounced neutrophilia with a more than twofold increase in neutrophil counts and a simultaneous 55% reduction in lymphocytes, reflecting the progression of systemic inflammation. A 55% reduction in monocyte counts was also observed, confirming their migration to the brain injury site. By day 7, neutrophil levels remained consistently elevated, while lymphocyte and monocyte counts remained reduced, with monocyte reduction reaching 71% compared to the control group. Overall, our program demonstrated results comparable to expert annotations, reflecting the general dynamics of peripheral blood cell composition changes in TBI.

The model’s F1-scores (0.75 for neutrophils, 0.69 for lymphocytes, 0.61 for monocytes, 0.43 for eosinophils) and mAP of 0.55 reflect robust performance for common cell types, aligning with the observed neutrophilia, lymphopenia, and monocytopenia post-TBI. Notably, the model detected a nearly twofold increase in platelet counts on day 1 post-TBI, potentially indicating early hemostatic activation, a finding less pronounced in manual counts. This suggests that automated analysis may capture subtle changes missed by manual methods, enhancing sensitivity to dynamic hematological shifts. However, the model’s performance was limited for rare cell types, such as eosinophils and hypersegmented neutrophils, due to significant class imbalance (e.g., hyperchromic erythrocytes: 3.9% of annotations; eosinophils: <0.5%). This necessitated grouping neutrophil subpopulations (segmented, banded, hypersegmented) and erythrocyte types (normal, schistocytes, hyperchromic) into single categories, as subclassification accuracy was below 0.60 (F1-score).

Future improvements will focus on addressing class imbalance and improving subclassification accuracy. Oversampling techniques, such as generative adversarial networks (GANs), will increase representation of rare cell types. Expanding the dataset to include at least 500 annotated instances per rare morphotype will enhance training robustness. Additionally, implementing ensemble methods or multi-stage detection pipelines may boost accuracy for rare populations, targeting F1-scores above 0.85 for all cell types.

The analysis of the relationship between Cx43 expression in brain tissue and leukocyte subpopulations in peripheral blood after TBI revealed that monocytes showed the strongest association with Cx43, whereas neutrophils and lymphocytes played a less prominent role. Linear regression indicated that the model for monocytes explained 75.4% of the variability in Cx43 expression, with high statistical significance and a strong positive contribution of monocytes. This was further confirmed by correlation analysis, which demonstrated a strong positive association between normalized monocyte levels and Cx43 expression. These findings are consistent with leukocyte profile data, where monocyte levels in the experimental group decreased during the early stages after TBI but returned to control values by day 7. It is likely that monocytes migrate to the site of traumatic brain injury [65], where they may modulate neuroinflammation and intercellular communication through Cx43-associated GJs. The strong correlation and regression results suggest that even with reduced monocyte levels in peripheral blood, their activity or local role within brain tissue may enhance Cx43 expression, in line with the established role of monocytes in neuroinflammation and reparative processes after TBI [65].

In contrast to monocytes, the model for neutrophils did not reach statistical significance, despite their pronounced increase in peripheral blood, particularly at day 3 post-TBI. The lack of a significant correlation or regression link between neutrophils and Cx43 may be explained by the temporal mismatch between the peak of neutrophil response at day 3 and the increase in Cx43 expression, which is thought to peak at day 7 post-TBI. Neutrophils are known to play a more prominent role in early inflammation [66], but their impact on Cx43 appears limited, possibly due to restricted migration into the brain or alternative mechanisms of action. Experimental data from CNS injury models indicate that transient activation of Cx43 contributes to tissue damage and astrocyte activation, whereas suppression of Cx43 reduces neutrophil recruitment and mitigates secondary injury, highlighting the complex interplay between Cx43 and the immune system [67].

The model for lymphocytes demonstrated only moderate explanatory power, with neither time nor lymphocyte levels emerging as significant predictors. This was corroborated by correlation analysis, which showed a moderate negative association only in nonparametric tests. These findings are consistent with leukocyte profile data showing a marked reduction in lymphocytes in the experimental group, particularly at day 3. The negative correlation with Cx43 may suggest that lymphocyte reduction is associated with enhanced inflammatory processes mediated by Cx43; however, this link appears less robust, potentially due to nonlinearity or the small sample size. The correlation between lymphocytes and neutrophils revealed a moderate negative association, consistent with their opposite dynamics: increasing neutrophils accompanied by decreasing lymphocytes, reflecting a shift in the immune response toward innate immunity. The absence of significant correlations between monocytes and other leukocyte subpopulations underscores their unique role in the context of TBI and Cx43 regulation.

Taken together, these results highlight the pivotal role of monocytes in regulating Cx43 expression, most likely through mechanisms linked to neuroinflammation or regeneration of injured neural tissue. In contrast, neutrophils and lymphocytes appear to exert a weaker influence on processes modulating Cx43 expression, possibly due to lower involvement in post-traumatic brain events or differences in temporal dynamics.

Additionally, automated analysis was used to assess changes in platelet and erythrocyte counts. Notably, platelet and erythrocyte levels may correlate with TBI severity and serve as prognostic markers of outcomes in this condition [68]. Our study showed a significant, nearly twofold increase in platelet levels within 24 h post-TBI, likely associated with the activation of hemostatic mechanisms and a compensatory response to vascular damage in TBI. The literature reports that TBI can cause elevated platelet levels [69], although some studies note platelet dysfunction within 6–48 h post-injury [70]. Other authors report a decrease in platelet counts in the context of TBI [71]. However, by day 7 post-injury, platelet levels decreased by 58%, indicating their recruitment in the ongoing inflammatory process. The reduction in erythrocyte counts on day 1, followed by a reverse trend by day 7 relative to the control, may be driven by adaptive mechanisms restoring oxygen transport function. Eosinophils were nearly undetectable throughout the experiment, confirming their minimal role in the systemic inflammatory response in TBI.

It is also known that TBI is accompanied by pronounced metabolic disturbances, including hypoxia, lactate accumulation, and reduced tissue buffering capacity, leading to a significant pH shift toward acidity [72]. Particularly severe acidification is observed in the immediate injury zone, where pH levels may drop to critical values around 6.5 [73]. This alteration in acid-base balance becomes a key factor triggering conformational reorganization of various molecules, including Cx43, whose structure and function may acquire a pathological character [74]. To confirm pH-dependent conformational reorganization of Cx43, molecular dynamics modeling of Cx43 under low pH conditions was conducted.

It was demonstrated that, among the three histidines present in Cx43 at positions 142, 194, and 331, only His142 participates in stable pH-dependent inter-residue interactions. Under physiological conditions, His142 predominantly faces Glu103 with its unshared electron pair and does not form hydrogen bonds with neighboring residues. However, under modeled conditions corresponding to ischemic acidosis with a pH of 6.5, protonated His142 begins to form a stable hydrogen bond with Glu103. This interaction stabilizes a specific conformation not observed under normal physiological conditions, indicating a pH-dependent mechanism of Cx43 structural reorganization. This acid-response model aligns with earlier data on the involvement of the L2 domain (amino acids 119–144 of the intracellular loop CL of Cx43) in pH-dependent GJ closure [75]. It was shown that at a pH of 6.5, the interaction between the L2 domain and the C-terminal domain (CT) of Cx43 is enhanced, likely reflecting an internal rearrangement of Cx43 that promotes channel closure [76,77]. According to the “ball-and-chain” model, the CT domain acts as a flexible gating component that, at low pH, binds to the L2 region, forming a closed channel configuration [78]. It is worth noting that this local change in Cx43, from the perspective of global RMSD metrics, is minimal. Nevertheless, pH-dependent protonation of specific residues, including His142, and domain-specific intramolecular interactions (CT–L2) may collectively ensure Cx43’s sensitivity to acid-base balance changes and contribute to fine-tuning its physiological and pathological activity.

Our hypothesis regarding the role of the interaction between protonated His142 and Glu103 in Cx43 function is supported by the known E103K mutation (COSV99246892), where negatively charged Glu is replaced by positively charged lysine (Lys). The significance of the E103K mutation in the connexin family is evidenced, for example, by the Glu-to-Lys substitution in Cx39.9, which disrupts electrical coupling between slow muscle fibers, manifesting as motor impairment [79]. This analogy fits well with our concept, mimicking Glu behavior during ischemia. For instance, the introduction of a positive charge at position 103 creates electrostatic attraction to the unprotonated aromatic nitrogen of His142, where a negative charge is localized due to an unshared electron pair. Likely, this mutation stabilizes an atypical Cx43 structure, similarly to that formed at low pH but in non-ischemic conditions. The molecular structure of Cx43 with the E103K mutation adopts a pathological conformation analogous to that seen in acute acidosis developing in damaged neural tissue during TBI.

It is also noteworthy that the E103K mutation is recognized as pathogenic, and its consideration in a low-pH model may serve as a natural anomaly confirming the fundamental importance of the local interaction between His142 and the amino acid residue at position 103 for normal Cx43 function. Protonation of His142, occurring during TBI-induced acidosis, effectively mimics this mutation, as both cases result in an anomalous hydrogen bond between the described amino acid residues. The pH-dependent interaction mechanism of protonated His142-Glu103 in the context of TBI-induced acidosis may be associated with severe neurotoxic effects, manifested through disrupted intercellular communication, hemichannel hyperactivation, ion leakage, excitatory mediators, and other synthesis and catabolism products, ultimately leading to the spread of secondary injury zones with progressive neuronal and glial cell death.

The modeling results not only describe conformational changes in Cx43 at critically low pH induced by TBI but also reveal a subtle structural mechanism operating at the level of hydrogen bonding between protonated His142 and Glu103. Additionally, the high consistency of our modeled data with the molecular mechanism of the E103K mutation allows it to be considered a model of persistent activation of an ischemia-induced conformation, which may be applicable in developing therapeutic strategies aimed at stabilizing the physiological state of Cx43.

## 4. Materials and Methods

### 4.1. Animals and Ethical Approval

Adult male CD-1 mice, aged 14–15 weeks and weighing 20–25 g, were used to study traumatic brain injuries. The animals were housed in groups of 6–7 in spacious cages with unlimited access to food and water. The facilities maintained a temperature of 22–25 °C, with a ventilation system providing 18 air changes per hour, ensuring comfortable conditions for their well-being.

All experiments were conducted in strict compliance with international, national, and institutional standards for the ethical treatment of laboratory animals. The studies adhered to the requirements of EU Directive 86/609/EEC (https://eur-lex.europa.eu/eli/dir/1986/609/oj, accessed on 28 April 2025) of 24 November 1986, as well as Russian regulations, including the “Rules of Good Laboratory Practice” (Order of the Ministry of Health of the Russian Federation No. 708n of 23 August 2010, https://normativ.kontur.ru/document?moduleId=1&documentId=165691, accessed on 5 June 2025) and GOST 33215–2014 (https://meganorm.ru/Index2/1/4293757/4293757886.htm, accessed on 12 June 2025), which governs the conditions of animal housing and experimental procedures. The research protocol No. 2 was approved by the Bioethics Committee of Don State Technical University on 17 February 2020, confirming its compliance with high ethical and scientific standards.

### 4.2. Objects and Procedure

To replicate severe TBI, an original model developed by the authors was used. Anesthesia was administered according to the following protocol: a mixture of Xyla (0.2 mL/kg, 2% xylazine hydrochloride solution; manufacturer: Interchemie Werken “de Adelaar” BV, Venray, Netherlands) and Zoletil (15 mg/kg, a combination of tiletamine and zolazepam hydrochlorides; Virbac, Carros, France) was injected intramuscularly. The depth of anesthesia was confirmed by the absence of response to painful stimuli and suppression of the corneal reflex.

Before surgical intervention, the fur on the mouse’s head was shaved, and the skin was thoroughly disinfected. The animal was secured in a specialized apparatus for trauma modeling. A longitudinal incision was made along the midline of the skull to expose the bone. The mouse’s head was fixed, and a 3 mm diameter hole was drilled using a dental drill. Through this hole, a 150 g metal rod with a 2 mm diameter and 3 mm long tip was dropped from a height of 1 cm. The impact point was determined by coordinates: 2 mm dorsal to the bregma and 1 mm lateral to the midline, corresponding to the parietal cortex. After the injury was inflicted, the hole was irrigated with saline, sealed with bone wax, and the wound was sutured.

### 4.3. Confocal and Fluorescent Microscopy

To investigate the localization of Cx43 in the mouse brain 24 h and 7 days post-TBI, the following methodological approach was employed. Animals were anesthetized, and perfusion was performed through the right ventricle of the heart with a 4% paraformaldehyde (PFA) solution. To ensure uniform PFA penetration, the animals were fixed upside down for 2 h. Additionally, the extracted brain was further incubated in 4% PFA for 12 h to complete fixation. Subsequently, a 0.4 cm thick frontal section, including the necrosis zone caused by TBI, was prepared. Thin sections, approximately 20 µm thick, were obtained using a Leica VT 1000 S vibratome (Leica Biosystems, Nussloch, Germany). These sections were sequentially incubated in 15% and 30% sucrose solutions for 1 h each for cryoprotection and then frozen at −80 °C.

Sections were washed in phosphate-buffered saline (PBS) and treated with a blocking solution containing 5% bovine serum albumin (BSA, Sisco Research Laboratories Pvt. Ltd., Mumbai, India) and 0.3% Triton X-100 (Sisco Research Laboratories Pvt. Ltd., Mumbai, India) for 1 h at room temperature to minimize non-specific antibody binding. The sections were then incubated for 48 h at 4 °C with primary antibodies: rabbit anti-Cx43 (1:100; E-AB-70097; Elabscience Biotechnology Inc., Houston, TX, USA) and mouse anti-neuron-specific nuclear protein NeuN (1:1000; FNab10266, FineTest, Wuhan, China) or astrocyte marker GFAP (1:1000, SAB4200571, Sigma-Aldrich, St. Louis, MO, USA). After multiple PBS washes, the sections were treated with secondary antibodies: for confocal microscopy, anti-rabbit IgG (H+L) Abberior STAR 635P (1:500, Abberior GmbH, Göttingen, Germany) and anti-mouse IgG (H+L) Abberior STAR 580 (1:500, Abberior GmbH, Göttingen, Germany) were used; for fluorescent microscopy, rabbit antibodies conjugated with Alexa Fluor 488 (1:500; ab150077, Abcam, Cambridge, United Kingdom) and mouse antibodies conjugated with Alexa Fluor 555 (1:500; ab150114, Abcam, Cambridge, United Kingdom) were applied.

Negative controls were performed without primary antibodies. Neuronal and glial cell nuclei were stained using Sytox Green Stain (ThermoFisher Scientific, Waltham, MA, USA) at a 1:1000 dilution in PBS for confocal microscopy or Hoechst 33,342 for fluorescent microscopy. Sections were incubated with the dye for 20–30 min at room temperature in the dark to ensure specific nuclear staining. After staining, sections were washed three times in PBS to remove excess dye and reduce background signal. Samples were then placed in an anti-photobleaching medium (Abberior GmbH, Göttingen, Germany) to protect fluorescent signals from degradation during prolonged exposure and covered with a coverslip to prevent drying and optimize visualization.

The study was conducted using an inverted confocal laser scanning microscope Abberior Facility Line (Abberior Instruments GmbH, Germany), which provides high resolution for analyzing cellular structures. For 3D model creation, Z-scanning was performed with a step size of 200 nm and a pixel size of 40 nm. Three-dimensional image reconstruction was carried out using ImageJ software (version 1.54j, National Institutes of Health, Bethesda, MD, USA). For fluorescent microscopy, an Olympus BX53 microscope (Olympus Corporation, Tokyo, Japan) equipped with a high-resolution digital camera (EXCCD01400KPA, Hangzhou ToupTek Photonics Co., Ltd., Hangzhou, China) was used.

For quantitative assessment of Cx43 expression levels in fluorescent microscopy images, ImageJ software (version 1.54j, National Institutes of Health, Bethesda, MD, USA) was employed. A rectangular region encompassing the entire field of view was selected for each image, and the mean fluorescence intensity was measured. Similarly, background intensity was measured in an area free of specific signals. The background intensity was subtracted from the mean signal intensity, and the result was normalized to the background using the following formula:

$$Relative\;Fluorescence\;Intensity(\%)=\left(\frac{{Mean}_{signal}{-Mean}_{background}}{{Mean}_{background}}\right)\times 100$$

The obtained values were expressed as percentages relative to background intensity and used for statistical analysis.

Colocalization of NeuN and Hoechst was also evaluated using ImageJ version 1.51r (http://rsb.info.nih.gov/ij/, accessed on 10 June 2017) with the JACoP plugin. The M1 coefficient was calculated to quantitatively assess the degree of signal overlap.

### 4.4. Transmission Electron Microscopy

To study the ultrastructure of mouse brain tissue after TBI, fragments of the parietal cortex, including both the injured area and corresponding regions of the uninjured hemisphere, were processed as follows. Samples were fixed in a 2.5% glutaraldehyde solution (Aurion, Eugene, OR, USA) to preserve cellular architecture. They were then washed in phosphate-buffered saline (PBS) to remove residual fixative and post-fixed in a 1% osmium tetroxide (OsO_4_) solution in phosphate buffer for 1.5 h to stabilize lipid structures.

For embedding in epoxy resin, samples were dehydrated by sequential incubation in ethanol solutions of increasing concentration (from 50% to absolute ethanol). This was followed by three stages of treatment with propylene oxide to facilitate infiltration, after which the tissues were embedded in Epon-812-based epoxy resin, forming a durable matrix for sectioning.

Semi-thin and ultra-thin sections were prepared using an EM UC 7 ultramicrotome (Leica, Wetzlar, Hesse, Germany) with an Ultra 45° diamond knife (Diatome, Biel/Bienne, Canton of Bern, Switzerland). Ultra-thin sections were contrasted with uranyl acetate and lead citrate solutions to enhance visualization of intracellular organelles and membranes.

Ultrastructural analysis was performed using a JEM-1011 transmission electron microscope (Jeol, Akishima, Tokyo, Japan) at an accelerating voltage of 80 kV, which provided high-resolution images for detailed examination of changes in neurons and glial cells post-TBI.

For objective and standardized morphological evaluation, a semi-quantitative approach was employed using a developed scale based on several key ultrastructural criteria. The degree of pathological changes in each image was assessed based on eight parameters: integrity of cell membranes, mitochondrial condition, myelin sheath structure, organization of intracellular compartments, presence of vacuoles, nuclear changes, presence of autophagosomes and lysosomes, and signs of microglial activation. Each criterion was scored on a four-point scale: 0—no pathological changes, 1—mild changes, 2—moderate, 3—severe, 4—critical. The total score for each image reflected the severity of pathological changes and could range from 0 to 32.

In cases where pronounced pathological changes prevented reliable identification of specific morphological elements, the criterion was assigned the maximum score.

### 4.5. Preparation of Blood Smears Using Romanowsky-Giemsa Staining

Blood smear preparation using the Romanowsky-Giemsa method followed this protocol. Peripheral blood was collected from mice on days 1 and 7 post-TBI, and thin smears were immediately prepared on degreased glass slides. Freshly prepared smears were air-dried at room temperature for approximately 15 min until fully dry. The dried smears were then covered with May–Grünwald solution and incubated for 3 min. Slides were gently rinsed under running water to remove excess fixative and air-dried again for 15 min.

Before main staining, a fresh working solution of Romanowsky-Giemsa dye was prepared by diluting the concentrated azure–eosin solution in PBS (pH 6.4–6.8) at a ratio of 1 part dye to 10 parts buffer. Fixed and dried smears were evenly covered with the diluted Romanowsky-Giemsa dye and stained for 40 min at room temperature. The preparations were then thoroughly rinsed with running water to remove background dye and buffer residues and allowed to air-dry in a vertical position.

Microscopic analysis of the prepared smears was conducted using immersion oil at 40× objective magnification to evaluate the morphology of peripheral blood cells in mice at the selected post-traumatic time points.

### 4.6. Molecular Dynamics Simulation

Molecular dynamics (MD) simulation was performed using GROMACS 2025.1 [80]. The starting geometry for Cx43 was predicted using the Boltz-2 algorithm [81]. The packmol-memgen utility from AmberTools24 [82] was used to generate a model of this protein within a lipid bilayer. The bilayer composition was designed to mimic a neuronal membrane, using standard lipids from the amber.lipids21 force field without extensions. The lipid composition and ratio for the inner//outer leaflet were as follows: “DPPC:POPE:OSM:DPPS:POPI:POPA:PAPI3:CHL1//DPPC:POPE:OSM:CHL1—ratio 129:23:14:93:48:4:13:422//241:13:58:443”. To investigate Cx43 structural changes in TBI, three models were studied: native (pH 7.0), acidosis (pH 6.5), and acidosis with Ca^2+^ ions. Using the tleap utility from AmberTools24, these models were prepared in a water box with K^+^ and Cl^−^ counterions at a concentration of 0.14 M. The Cx43 model under acidosis conditions was prepared using the H++ resource [82].

MD simulations followed a unified protocol suitable for proteins, nucleic acids, and protein–nucleic acid complexes in the amberff19SB and lipids21 force fields. The tip3p model was used for water and ions. Long-range electrostatic interactions were modeled using the PME method, and van der Waals forces were considered at a distance of 1.2 nm from the atom. Simulations were conducted at 310 K. The first stage involved stepwise (steepest descent) geometry minimization until maximum forces on atoms reached 500 kJ/mol·nm, followed by further optimization using the l-bfgs method. The second stage involved system equilibration for temperature and pressure. The resulting geometry underwent MD simulation for 200 ns. RMSD changes and final model geometries were analyzed to evaluate the simulation results.

### 4.7. Computer Vision Model

The computer vision neural network model was developed following a standard machine learning pipeline. Microscopic blood smear images were acquired over three independent experimental days, with approximately 60 fields of view captured per day, resulting in a total dataset of 180 images. Each image underwent single-cell level annotation into 18 morphological categories, encompassing both common cell types (e.g., normal erythrocyte, echinocyte, hyperchromic erythrocyte) and rare morphological variants (e.g., monocyte, eosinophil, large granular lymphocyte). The dataset exhibited significant class imbalance, with the most frequent class (hyperchromic erythrocytes) comprising approximately 3.9% of the total annotated objects.

Initially, PNG format images were annotated with bounding boxes using the https://www.makesense.ai/platform (accessed on 10 June 2025) and exported in COCO JSON format by one operator. Subsequently, both images and annotations underwent data augmentation using transformations that are safe for microscopic imaging to simulate realistic imaging conditions: rotations at 90°, 180°, and 270°, and brightness adjustment within the range of [0.9, 1.15]. The dataset was partitioned into training, validation, and test sets with a 70/20/10 ratio, with separate JSON files generated for the training and validation sets. Each subset was stratified to ensure proportional representation from both experimental and control datasets, as well as balanced distribution across all three experimental days, preventing data leakage between sets.

For model training, the AutoGluon framework was employed with the pre-configured “dino-best” preset, corresponding to the DINO-SwinL architecture. Training was conducted over 97 epochs with batch_size = 2 and max_epoch = 20 parameters on an NVIDIA RTX 4090 graphics card. Due to video memory limitations, both training and validation datasets were randomly shuffled and divided into five equal-sized subsets. The model underwent sequential training on each subset, with weights obtained from the previous stage serving as initial parameters for the subsequent stage, enabling gradual training on the complete dataset without exceeding available computational resources. Model performance evaluation was conducted using the model automatically saved by AutoGluon as the best performer according to internal validation metrics.

### 4.8. Statistical Analysis

For the statistical analysis of data obtained in the experiments on Cx43 expression, NeuN/Hoechst colocalization, ultrastructural changes in the cerebral cortex, and leukocyte composition in peripheral blood after TBI, the following methods were applied. Mean fluorescence intensity of Cx43 and the M1 colocalization coefficient (NeuN/Hoechst) were analyzed using one-way analysis of variance (ANOVA) with Tukey’s post hoc test for group and time point comparisons. Ultrastructural alterations of the cerebral cortex, assessed by transmission electron microscopy, were semi-quantitatively evaluated across eight morphological criteria using ANOVA with Tukey’s post hoc test.

The leukocyte composition of peripheral blood was determined at 1, 3, and 7 days after TBI using Romanowsky-Giemsa-stained smears examined by microscopy, as well as automated cell detection based on computer vision. The dynamics of leukocyte profile changes were analyzed using repeated-measures ANOVA with Holm’s post hoc test for comparisons between experimental and control groups. Data normality was verified with the Shapiro–Wilk test, and homogeneity of variances with the Brown–Forsythe test. In cases of violated assumptions of normality or homogeneity, the nonparametric Kruskal–Wallis test with Dunn’s post hoc test was applied.

To examine associations between normalized Cx43 levels and leukocyte subpopulations, linear regression and correlation analyses were performed. Experimental group data (n = 12, with 6 values each for day 1 and day 7) were normalized by dividing Cx43 and leukocyte levels by the respective mean values of the control group for each time point. Linear regression was conducted separately for each leukocyte subpopulation, with normalized Cx43 expression as the dependent variable, and predictors including time (day 1 vs. day 7, treated as a categorical factor with day 1 as reference) and the normalized level of the respective subpopulation. For each model, the coefficient of determination (R^2^), adjusted R^2^, F-statistic, and *p*-values were calculated to assess overall model significance, along with unstandardized and standardized regression coefficients (β) and their corresponding *p*-values.

Correlation analysis was performed using Pearson, Spearman, and Kendall methods to evaluate pairwise associations between normalized Cx43 levels and each leukocyte subpopulation, as well as between subpopulations, with calculation of correlation coefficients (r, ρ, τ) and associated *p*-values. Regression assumptions, including residual normality and linearity, were checked using Q–Q plots and residual visualizations.

All statistical analyses were carried out using JASP software (version 0.19.1, University of Amsterdam, Amsterdam, The Netherlands) and SigmaPlot (version 12.5, Systat Software Inc., San Jose, CA, USA). All analyses were performed blinded. Statistical significance was set at *p* < 0.05. Data are presented as mean ± standard error of the mean (M ± SEM), with a sample size of n = 6 for each time point and group.

## 5. Conclusions

The obtained results demonstrated the dynamic heterogeneity of Cx43 expression, closely associated with morphofunctional alterations of neurons and astrocytes in the cerebral cortex at different stages after TBI. In uninjured tissue, Cx43 expression showed a uniform distribution, reflecting stable intercellular communication, whereas after TBI its level sharply decreased within 24 h, coinciding with destructive changes in nuclear structures and reduced NeuN/Hoechst colocalization, indicating the activation of apoptosis and necrosis. This decline was likely associated with the loss of astrocytes, the main source of Cx43, as well as its degradation and possibly the influence of pro-inflammatory cytokines, whose concentrations increase in the acute phase of injury.

By day 7, Cx43 expression significantly increased, acquiring a heterogeneous pattern with aggregate formation, which correlated with progressive reactive astrogliosis, glial scar formation, and further NeuN nuclear loss, reflecting the development of chronic neurodegenerative processes. Ultrastructural analysis confirmed these findings, revealing progressive damage to membranes, organelles, mitochondria, and myelin sheaths, reaching a critical level by day 7, along with accumulation of amoeboid microglial cells involved in phagocytosis and tissue remodeling.

The systemic inflammatory response, reflected in leukocyte dynamics, was characterized by an early rise in segmented neutrophils, peaking at day 3 and remaining elevated at day 7, indicative of acute and chronic inflammatory activity. In contrast, monocytes decreased in the first days, likely due to migration into the lesion site, while lymphocytes exhibited pronounced depression. Automated machine learning-based analysis confirmed these changes, refining the dynamics of platelets and erythrocytes, with platelet elevation in the acute phase followed by a decline at day 7, underscoring hemostasis activation and the potential of the platform to detect subtle shifts overlooked by manual methods, despite its limitations in rare cell detection that require improvement through oversampling and ensemble approaches.

The analysis of Cx43 associations with leukocyte subpopulations revealed a strong positive correlation with monocytes, confirmed by regression and correlation analyses, consistent with their migration into the brain and role in neuroinflammation, in contrast to the weak or absent correlation with neutrophils and lymphocytes, where temporal asynchrony may play a key role.

Moreover, molecular dynamics simulations demonstrated a pH-dependent conformational reorganization of Cx43 under TBI-induced acidosis, with protonation of His142 and the formation of a hydrogen bond with Glu103, resembling the pathogenic E103K mutation. This led to stabilization of an abnormal structure and potential disruption of GJ functions, exacerbating secondary damage. These mechanisms, including interactions between L2- and CT-domains, highlight acidosis as a critical factor of neurotoxicity, suggesting novel therapeutic targets.

Overall, the findings reveal complex mechanisms of Cx43 regulation in TBI, emphasizing its dual role in inflammation and regeneration, as well as the significance of monocytes and pH-dependent changes. This expands fundamental understandings of neuroglial interactions under traumatic stress and opens new perspectives for developing therapeutic strategies through modulation of Cx43 levels and immune response regulation.

Based on the comprehensive study that combined molecular–biological, morphological, and in silico analyses with machine learning approaches, the following conclusions were formulated:

1. TBI induces dynamic changes in Cx43 expression in the cerebral cortex, characterized by a decrease in the acute phase (24 h) and a significant increase with the formation of protein aggregates by day 7, which is associated with reactive astrogliosis.
2. Ultrastructural analysis reveals progressive damage to neurons and glia after TBI, including degradation of mitochondria, myelin sheaths, and synapses, reaching a critical level by day 7.
3. Peripheral blood analysis demonstrates persistent neutrophilia, lymphopenia, and reduced monocyte levels, reflecting systemic inflammatory response and immunodepression, confirmed by automated analysis using ML.
4. Linear regression and correlation analyses identify a strong positive association between normalized monocyte levels and Cx43 expression, a moderate negative correlation with lymphocytes, and no significant correlation with neutrophils.
5. Molecular dynamics simulations demonstrate a pH-dependent conformational reorganization of Cx43 during post-traumatic acidosis, driven by the interaction of protonated His142 with Glu103, mimicking the effect of the pathogenic E103K mutation and contributing to neurotoxic outcomes.
6. The developed ML-based platform for blood analysis effectively detects subtle hematological alterations, including early platelet elevation, and demonstrates high accuracy for major cell types, but requires optimization for rare subpopulations such as eosinophils.

## Figures and Tables

**Table 1:** Mean percentage of leukocyte subpopulations in peripheral blood of mice after TBI, presented as M ± SEM, calculated as a proportion of the total leukocyte count.

	1 Day		3 Days		7 Days	
Cells	Control	Experiment	Control	Experiment	Control	Experiment
Segmented neutrophils	35.54 ± 2.83	49.79 ± 1.50	33.05 ± 6.41	62.65 ± 3.41	41.41 ± 2.73	51.35 ± 3.66
Band neutrophils	12.60 ± 2.69	11.23 ± 1.19	8.97 ± 2.14	1.80 ± 0.92	3.32 ± 0.77	5.12 ± 1.73
Hypersegmented neutrophils	0.00 ± 0.00	0.00 ± 0.00	2.29 ± 1.11	3.22 ± 1.81	0.79 ± 0.79	1.60 ± 1.22
Monocytes	15.83 ± 1.75	7.54 ± 0.91	17.63 ± 4.00	12.34 ± 2.39	9.20 ± 1.28	8.62 ± 0.75
Lymphocytes	35.55 ± 3.53	31.44 ± 0.71	37.30 ± 2.74	17.79 ± 2.04	44.48 ± 2.99	32.29 ± 1.66
Eosinophils	0.48 ± 0.48	0.00 ± 0.00	0.00 ± 0.00	2.21 ± 1.40	0.79 ± 0.79	1.02 ± 0.67

**Table 2:** Results of linear regression analyses examining the relationship between Cx43 expression and levels of leukocyte subsets in peripheral blood on days 1 and 7 post-traumatic brain injury. Models include time and the respective leukocyte subset as predictors. Reported are R^2^, adjusted R^2^, F-statistics, *p*-values for the model (ANOVA), and coefficients (β), along with standardized β for continuous predictors.

Leukocyte Subset	R^2^ (Adjusted R^2^)	F (df)	*p* (ANOVA)	Predictor	Unstandardized β	Standardized β	t	*p*
Neutrophils	0.468 (0.350)	3.965 (2,9)	0.058	Time	1.052	-	2.734	0.023
				Neutrophils	0.519	0.116	0.474	0.647
Monocytes	0.754 (0.700)	13.83 (2,9)	0.002	Time	0.397	-	1.204	0.259
				Monocytes	1.182	0.692	3.313	0.009
Lymphocytes	0.527 (0.422)	5.018 (2,9)	0.034	Time	0.836	-	2.030	0.073
				Lymphocytes	−1.207	−0.305	−1.171	0.271

**Table 3:** Results of correlation analysis using Pearson, Spearman, and Kendall methods for normalized levels of Cx43 and leukocyte subsets, specifically neutrophils, monocytes, and lymphocytes, in peripheral blood on days 1 and 7 following TBI, as well as correlations between leukocyte subsets. The table reports correlation coefficients r, ρ, and τ along with their corresponding *p*-values. A strong positive correlation between monocytes and Cx43 underscores their critical role in post-traumatic processes, whereas lymphocytes exhibit a moderate negative correlation, and neutrophils show no significant correlation.

Variables	Pearson (r)	*p* (Pearson)	Spearman (ρ)	*p* (Spearman)	Kendall (τ)	*p* (Kendall)
Monocytes—Cx43	0.846	<0.001	0.727	0.010	0.485	0.031
Lymphocytes—Cx43	−0.557	0.060	−0.692	0.016	−0.515	0.021
Neutrophils—Cx43	0.164	0.611	0.336	0.287	0.273	0.250
Lymphocytes—Monocytes	−0.426	0.168	−0.483	0.115	−0.303	0.197
Lymphocytes—Neutrophils	−0.608	0.036	−0.727	0.010	−0.576	0.009
Monocytes—Neutrophils	−0.203	0.527	−0.126	0.700	−0.061	0.841

**Table 4:** Mean percentage of leukocyte subpopulations in peripheral blood of mice after TBI, presented as M ± SEM, calculated as a proportion of the total leukocyte count and determined using a trained neural network model.

	1 Day		3 Days		7 Days	
Cells	Control	Experiment	Control	Experiment	Control	Experiment
Neutrophils	42.4 ± 6.8	62.3± 5.1	34.3 ± 6.6	70.8 ± 3.8	35.5 ± 4.3	60.0 ± 4.0
Monocytes	0.0 ± 0.0	3.6± 2.4	12.4 ± 8.1	5.6 ± 3.0	15.1 ± 2.0	4.3 ± 1.8
Lymphocytes	57.6 ± 6.8	34.1 ± 4.5	51.9 ± 8.8	23.6 ± 4.7	49.4 ± 6.4	35.7 ± 4.0
Eosinophils	0.0 ± 0.0	0.0 ± 0.0	1.4 ± 0.5	0.0 ± 0.0	0.0 ± 0.0	0.0 ± 0.0

## Data Availability

The original contributions presented in this study are included in the article/Supplementary Materials. Further inquiries can be directed to the corresponding author.

## Supplementary Materials

The following supporting information can be downloaded at: https://www.mdpi.com/article/10.3390/ijms26188855/s1.
